# Maternal e-cigarette use can disrupt postnatal blood-brain barrier (BBB) integrity and deteriorates motor, learning and memory function: influence of sex and age

**DOI:** 10.1186/s12987-023-00416-5

**Published:** 2023-03-10

**Authors:** Sabrina Rahman Archie, Ali Ehsan Sifat, Yong Zhang, Heidi Villalba, Sejal Sharma, Saeideh Nozohouri, Thomas J. Abbruscato

**Affiliations:** Department of Pharmaceutical Sciences, School of Pharmacy, Texas Tech University Health Sciences Center, Amarillo, TX 79106 USA

**Keywords:** Electronic cigarette, Maternal, Postnatal, Blood-brain barrier, Behavioral outcomes, Sex, Age

## Abstract

Electronic nicotine delivery systems (ENDS), also commonly known as electronic cigarettes (e-cigs) are considered in most cases as a safer alternative to tobacco smoking and therefore have become extremely popular among all age groups and sex. It is estimated that up to 15% of pregnant women are now using e-cigs in the US which keeps increasing at an alarming rate. Harmful effects of tobacco smoking during pregnancy are well documented for both pregnancy and postnatal health, however limited preclinical and clinical studies exist to evaluate the long-term effects of prenatal e-cig exposure on postnatal health. Therefore, the aim of our study is to evaluate the effect of maternal e-cig use on postnatal blood-brain barrier (BBB) integrity and behavioral outcomes of mice of varying age and sex. In this study, pregnant CD1 mice (E5) were exposed to e‐Cig vapor (2.4% nicotine) until postnatal day (PD) 7. Weight of the offspring was measured at PD0, PD7, PD15, PD30, PD45, PD60 and PD90. The expression of structural elements of the BBB, tight junction proteins (ZO-1, claudin-5, occludin), astrocytes (GFAP), pericytes (PDGFRβ) and the basement membrane (laminin α1, laminin α4), neuron specific marker (NeuN), water channel protein (AQP4) and glucose transporter (GLUT1) were analyzed in both male and female offspring using western blot and immunofluorescence. Estrous cycle was recorded by vaginal cytology method. Long‐term motor and cognitive functions were evaluated using open field test (OFT), novel object recognition test (NORT) and morris water maze test (MWMT) at adolescence (PD 40–45) and adult (PD 90–95) age. In our study, significantly reduced expression of tight junction proteins and astrocyte marker were observed in male and female offspring until PD 90 (P < 0.05). Additionally, prenatally e-cig exposed adolescent and adult offspring showed impaired locomotor, learning, and memory function compared to control offspring (P < 0.05). Our findings suggest that prenatal e-cig exposure induces long-term neurovascular changes of neonates by disrupting postnatal BBB integrity and worsening behavioral outcomes.

**Supplementary Information:**

The online version contains supplementary material available at 10.1186/s12987-023-00416-5.

## Introduction

Maternal smoking during pregnancy remains one of the most important modifiable risk factors for poor pregnancy outcomes in the U.S. as well as globally [1–4]. Maternal smoking not only adversely impacts maternal health but also results in poor fetal outcomes including but not limited to tobacco-induced abortions and stillbirths, low birth weight (LBW), sudden infant death syndrome (SIDS), preterm birth, neurological and cognitive delays, congenital disabilities, colic, asthma and atopic pregnancies [3–5]. Considering the impact on the cerebrovascular system, several studies have demonstrated the long-term neurotoxic effects of maternal smoking on neonatal/postnatal rodent offspring including hyperactivity, decreased learning and memory function, increased depression, altered brain development and enhanced hypoxic ischemic brain injury [6–13].

Electronic cigarette or e-cigarette (e-cig), commonly known as vaping is a battery powered electronic nicotine delivery systems (ENDS), which has become extremely popular among all age groups and sexes since it entered the US market in 2007 [6, 14]. E-cig usage is considered as a safer alternative to conventional tobacco smoke because of their reportedly lower levels of nicotine and potential carcinogens and has been proposed to be utilized as a smoking cessation tool and for recreational purposes [15–17]. Surprisingly, recent studies have reported that e-cig usage is also popular in women of child-bearing age and up to 15% of the pregnant women are now using e-cigs [17–19]. E-cigs mainly contain a solution of nicotine along with several additives including propylene glycol, vegetable glycerin, acrolein, formaldehyde, flavoring agents and other trace elements, some of which may be toxic for health including developing fetus and offspring [14, 20]. Although long-term toxic effects of prenatal tobacco smoking on postnatal health are well documented and well established, limited preclinical and clinical studies exist to evaluate the impact of maternal vaping on neonatal health and neurovascular effects. However, increasing evidence from animal studies have demonstrated that maternal vaping may also impact brain development of neonates and adolescents. It has been reported in clinical studies that e-cig use during pregnancy can impair fetal development through altered neurologic, pulmonary, and cardiovascular dysfunction [21]. Recent studies, including our lab, have shown that prenatal e-cig exposure is associated with several cerebrovascular and neurological dysfunctions including genetic/epigenetic alteration in developing brain, cognitive dysfunction, decreased brain glucose utilization and increased hypoxic-ischemic brain injury, alteration in neural regulators of energy homeostasis, and inflammation, disruption in brain excitatory/inhibitory neuron balance and calcium homeostasis, microglial cell death, alteration in gene expression in the frontal cortex and localized inflammation of the hippocampus [6, 14, 22–28]. A challenge and potential danger is that most of the e-cigs available in the market contain various concentrations of nicotine. As nicotine can cross the blood-placental barrier and accumulate in fetal blood, [29, 30] it is important to understand the neurologic effects of prenatal nicotine exposure to a developing fetus.

The blood-brain barrier (BBB) is the vital component of central nervous system (CNS) which plays a major role in maintaining the homeostasis of the brain microenvironment by controlling the passage of substances and regulating the trafficking of immune cells between the blood and the brain. BBB development starts during the fetal stage, and it is well constructed by the point of birth, especially for restriction of macromolecules and proteins. At the cellular level, the BBB consists of microvascular endothelial cells (EC) lining the luminal walls of brain microvessels alongside closely associated pericytes embedded within the basal membrane and surrounded by astrocytic end-feet processes. Studies have shown that BBB disruption has been associated with the onset and/or progression of major neurological disorders including ischemic stroke, multiple sclerosis, amyotrophic lateral sclerosis, traumatic brain injury, brain tumor, Alzheimer’s and Parkinson’s disease, epilepsy, edema and glaucoma [31]. Several preclinical studies, including our lab, have demonstrated that tobacco smoke and e-cig disrupt the BBB by decreased tight junction proteins, increased permeability [32–34], altered ion transporter expression and activity at BBB [35–37] and can induce oxidative stress and inflammation in brain [38] which may worsen brain injury and ischemic stroke outcomes [39]. Although there are several studies available demonstrating the neurotoxic impact of e-cig exposure on neurological and cerebrovascular systems, extensive research needs to be conducted to assess the long-term effect of maternal e-cig exposure on postnatal cerebrovascular dysfunction. To the best of our knowledge as of today, there is no such a study reported on the effect of maternal vaping on postnatal cerebrovascular disruption. Given the rapidly growing popularity of e-cig use in pregnant women and its potential toxic effect(s) on postnatal health, it is critical to conduct preclinical studies to evaluate the long-term effect of maternal e-cig exposure on postnatal brain health in different developmental ages and sex with understanding any underlying mechanisms. Therefore, the aim of this study is to evaluate the effect of maternal e-cig use during pregnancy on postnatal BBB integrity, motor, learning and memory function at different ages using an *in-vivo* mice model.

## Material and methods

### Animals and surgical procedures

**Fig. 1:**
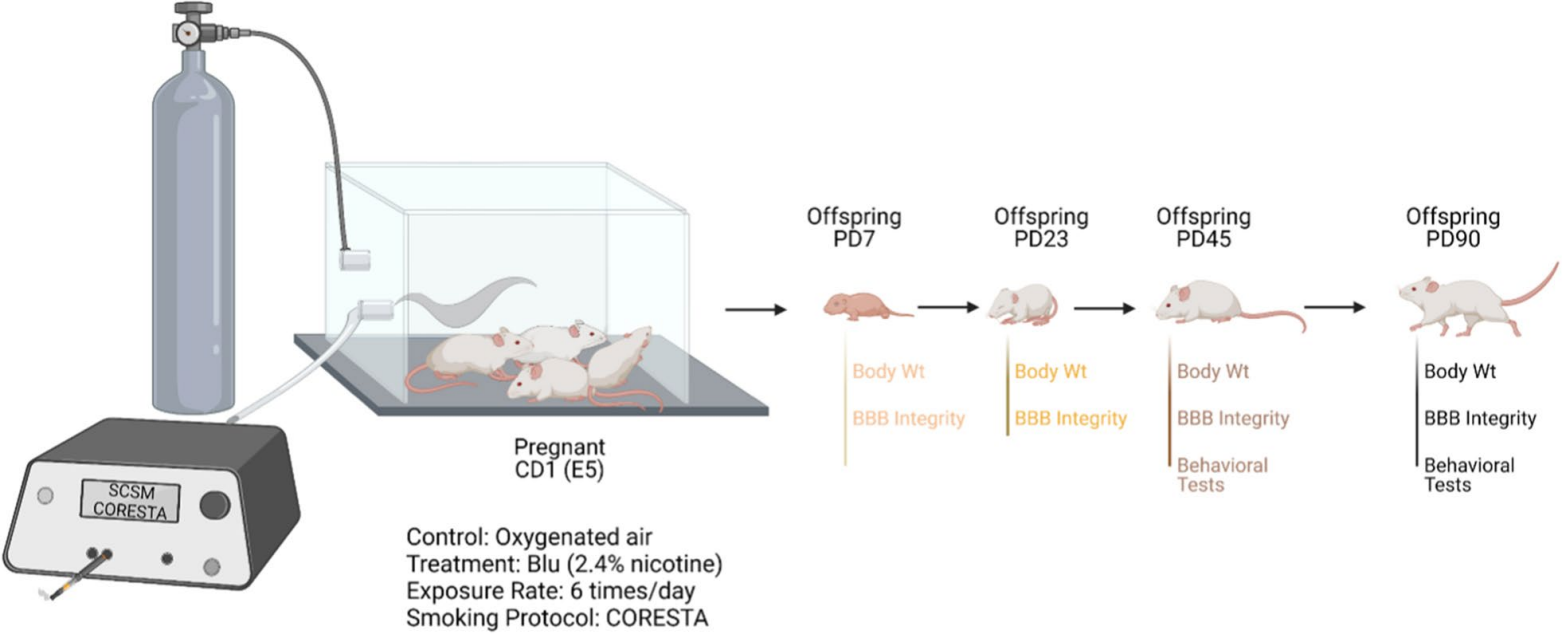
Study design. Pregnant CD1 were exposed to Blu e-cigarette from gestational day 5 (E5) to postnatal day 7 (PD7). At the end of the exposure, plasma nicotine and cotinine level were measured by LCMS/MS, and body weight was measured at PD7, PD23, PD45 and PD90. Mice were sacrificed and brain was extracted at every time point to evaluate blood-brain barrier (BBB) integrity by western blot and immunofluorescence. Open field test, novel object recognition test and morris water maze test were conducted at adolescent and adult time point to evaluate hyperactivity and learning-memory function

All studies were approved by the IACUC of Texas Tech University Health Sciences Center, Lubbock, Texas (IACUC protocol# 20026). Experiments were performed in accordance with relevant guidelines and regulations. Female CD1 pregnant mice (Charles River Laboratories, Inc., Wilmington, MA; Cat# CRL: 22, RRID: IMSR_CRL:22) and after delivery their offspring were kept under standardized light and dark conditions (12 h), humidity (70%), and temperature (22 °C). Pregnant mice were singly housed. Offspring were separated into male and female after weaning (postnatal day 21–22) and housed in a group of 2–5. They were given ad libitum access to food and water. Animal behavior was monitored daily to minimize animal suffering. We applied the following exclusion criteria to our experiments: severe weight loss, infections, or significant behavioral deficits (decreased mobility, seizures, lethargy). No animal was excluded from this study. The research design is depicted in Fig. 1. A total number of 176 (n = 16 for mother and n = 160 for offspring) mice were used to perform this study. All experiments were conducted in compliance with the ARRIVE guidelines.

### In-vivo e-cig vaping

Pregnant CD1 mice were exposed (via direct inhalation) to e-cig vapor containing 2.4% nicotine (Blu^™^, 24 mg/ml nicotine) mixed with oxygenated air or oxygenated air alone, 6 times/day; 1 cartridge/day from gestational day 5 (E5) until delivery, and it was continued after delivery until the pups were 7 days old. After birth, the pups would be exposed to nicotine via lactation [40, 41]. This exposure model was adopted following a study by Sifat et al. who investigated the effects of prenatal electronic cigarette exposure on offspring in mice model [6, 8]. In our study, Blu^™^ was used as this is one of the most popular e-cig brands still on the market, the cig-a-like structure fits well in our smoking apparatus, and there have been previously reported studies using Blu^™^ [6, 34, 37]. A modified CORESTA (Cooperation Centre for Scientific Research Relative to Tobacco) standard smoking protocol adapted to study e-cig exposure (27.5 ml puff depth volume, 3 s puff duration, 2 puffs per 60 s, 32 puffs/session) was followed in the laboratory. E-cig vapor was generated using a Single Cigarette Smoking Machines (SCSM, CH Technologies Inc.) following a previously published method used by our laboratory [6, 34, 37]. This method was developed to mimic the smoking behavior of a human chronic/heavy smoker/vaper and yields plasma levels of cotinine (111 ng/ml) which is in the range of blood cotinine level (30–250 ng/ml) found in other preclinical rodent models of chronic e-cig exposure [42, 43]. The smoking exposure was done between 9 a.m. and 2 p.m.

### Nicotine and cotinine level measurement by LCMS/MS in offspring plasma

Concentration of nicotine and its principal metabolite cotinine were measured from prenatally e-cig exposed mice plasma at PD7 by LCMS/MS analysis using Cotinine-d3 (MilliporeSigma, St. Louis, MO, USA) as an internal standard (IS) following a previously published method [44]. In brief, samples were prepared by protein precipitation of 25 µL mouse plasma using acetonitrile at 1:8 ratio. Mass Spectrometer was operated in positive polarity under the multiple reaction monitoring mode using electrospray ionization technique. The transitions of m/z 163.2 → 132.1, 177.2 → 98.0 and 180.2 → 101.2 were used to measure the nicotine, cotinine, and IS, respectively. The elution of nicotine (MilliporeSigma), cotinine (MilliporeSigma), and IS were at 1.89, 1.77, and 1.76 min, respectively. This was achieved with a mobile gradient phase consisting of 5 mM ammonium bicarbonate, acetonitrile, and methanol (3:1, v/v) at a 0.3 mL/min flow rate on a Kinetex EVO C18 column (Phenomenex, Torrance, CA, USA).

### Body weight measurement

Weight of the mother was measured before e-cig exposure (E5) and post-delivery. Litter size and litter weight were also counted and measured respectively. The weight of offspring was measured at several time points including PD7, PD15, PD30, PD45, PD60, and PD90 to evaluate the body growth and development. To measure the brain to body weight ratio, brain was weighed after extraction at PD7, PD23, PD45, PD90 and brain-to-body weight was measured.

### Western blot

Prenatally e-cig exposed, or control mice were sacrificed at each time points (PD7, PD23, PD45 and PD90) and brains were isolated. Brains were lysed using RIPA buffer to isolate protein lysate. Protein concentration of protein lysates were determined using a bicinchoninic acid (BCA) assay. Exactly 30 μg of protein from each sample was loaded and separated using a 10% Tris-glycine polyacrylamide precast gel (Bio-Rad Laboratories, Hercules, CA; Cat# 4568034). This method has been used previously to analyze Western blot immunoreactivity [6]. Protein samples were then transferred to a polyvinylidene difluoride membrane (Thermo Fisher; Cat# IPVH00010), and then membranes were incubated in blocking buffer (0.2% Tween-20 containing Tris-buffered saline (TBST) with 5% bovine serum albumin) to block the nonspecific protein bands for 2 h at room temperature. Membranes were incubated with rabbit polyclonal anti-ZO-1 antibody (1: 2000, Thermo Fisher; Cat# 40–2200), mouse monoclonal anti-claudin-5 antibody (1: 2000, Thermo Fisher; Cat# 35–2500), rabbit monoclonal anti-occludin antibody (1: 1000, Cell Signaling; Cat# E6B4R), rabbit polyclonal anti-laminin α1 antibody (1:2000, Thermo Fisher; Cat# PA1-16730), rabbit polyclonal anti-laminin α4 antibody (1:2000, Sigma; Cat# SAB4501719), rabbit monoclonal anti-GFAP antibody (1: 2000, Cell Signaling; Cat# DIF4Q), Rabbit monoclonal anti-PDGFRβ antibody (1: 1000, Cell Signaling; Cat# 28E1), mouse monoclonal anti-AQP4 antibody (1: 1000, Santa Cruz; Cat# 2), rabbit monoclonal anti-NeuN antibody (1: 1000, Cell Signaling; Cat# D3S3I), rabbit monoclonal anti-Glut-1 antibody (1: 2000, Cell Signaling; Cat# D3J3A) and mouse monoclonal anti-beta-actin antibody (1: 10000 MilliporeSigma; Cat# A5441) in TBST with 5% bovine serum albumin at 4 °C overnight. After 4 times washing with TBST for 15 min each cycle, membranes were incubated with anti-rabbit (Sigma Aldrich; Cat# GENA934- 1ML, RRID: AB_2722659) or anti-mouse (Sigma Aldrich; Cat# GENXA931-1ML, RRID: AB_772209) IgG-horseradish peroxidase secondary antibody (1:10000) in TBST with 5% bovine serum albumin for 2 h at room temperature. After 4 times of 15 min wash with TBST, the protein signals were detected by enhanced chemiluminescence-detecting reagents (Thermo Fisher; Cat# 34577) and visualized in X-ray films in the dark. The protein bands were quantified relative to beta-actin in Image J software.

### Immunofluorescence

Immunofluorescence staining was performed as previously described with modifications [45, 46]. Mice were euthanized by isoflurane overdose at each time point. The brains were sectioned at 20 µM of thickness, fixed with 4% paraformaldehyde (Thermo Fisher) for 15 min, then permeabilized with 0.1% Triton X-100 for 10 min. After washing with the phosphate-buffered saline (PBS) for 15 min, the sections were blocked for 1 h and incubated overnight with primary antibodies for ZO-1 (1:100, Thermo Fisher) claudin-5 (1: 100, Thermo Fisher) and GFAP (1:100, Cell Signaling), respectively. Alexa fluorescent secondary antibodies (Thermo Fisher) were used at 1:400 dilutions for 1 h. After counterstaining with 4′,6-diamidino-2-phenylindole (DAPI) for nucleus and washing with PBS, the sections were mounted with Permount (Thermo Fisher). The whole sections were scanned with a Leica Stellaris SP8 Falcon microscope (Leica Microsystem) and the images (20X magnitude) were captured with the same microscope. Mean total fluorescence intensity was calculated for each color channel and intensity of green color (ZO-1/GFAP) and red color (claudin-5) was expressed relative to blue color (DAPI). Cortex and hippocampus of both hemispheres of each brain section were used to evaluate the expression levels of ZO-1, claudin-5 and GFAP. To minimize the subjective bias, all images for ZO-1, claudin-5 and GFAP expression analysis were captured under the same microscopic parameter (laser power, pinhole size, exposure time) setting.

### Open field test (OFT)

OFT was performed to evaluate the locomotor activity of the prenatally e-cig exposed or control mice both male and female at PD45 (adolescent) and PD90 (adult) following our previously published study [47, 48]. Versamax software (Accuscan Instruments., Columbus, OH) was used to automatically calculate the total distance traveled by the mice. Briefly, mice were introduced to 16″ × 16″ unobstructed glass chamber and their activities were monitored and recorded for 1 h. The first 10 min of 1 h was excluded as the acclimatization period. All experiments were performed between 8 and 10 am. Fecal boli was counted for each mouse after completing of the OFT to measure stress/anxiety level following published literatures [49, 50].

### Novel object recognition test (NORT)

NORT was performed to evaluate short-term memory retention. It was done by a slight modification of previously published literature [51]. For habituation, each mouse was placed in a wooden box without any object for 10 min, 24 h before the test. On the testing day, mouse was placed in that same box containing two identical green round blocks for 5 min for the familiarization phase. After a 30 min interval, during the test phase one of the objects was replaced with an orange rectangular shaped object. The time spent by the mice exploring each object was recorded by video capture and analyzed. The results are presented as the discrimination index which is calculated by: (time exploring the novel object—time exploring the familiar object)/(time exploring the novel object + time exploring the familiar object). It is common rodent behavior for a mouse to explore a novel object over a familiar one. The premise for this test is that a mouse with a cognitive deficit will not be able to remember the old object during the test phase, therefore will spend a similar amount of time exploring each object. All experiments were performed between 10 a.m. and 12 p.m.

### Morris water maze test (MWMT)

MWMT was performed to assess spatial learning and memory function [52]. A circular tank of 4 ft diameter was filled with water and the water was made opaque by the addition of non-toxic blue paint. The temperature of the water was maintained at 22 °C. Spatial cues of various shapes (round, rectangle, square, triangle) and colors (red, yellow, green, blue) were equally spaced and placed around the tank. An escape platform positioned 1 cm below the surface of the water and mice were trained to locate it. This study is of 5 days: day 1–4 are trial days and day 5 is probe test day. On trial days, each mouse had 3 training trials per day separated by 1 h. In each trial, the mouse was placed in one of the 3 start locations which were equally spaced around the perimeter of the tank. Start location was changed in each trial. The mouse was allowed to swim for 60 s or until it reached the platform. If the mouse could not locate the platform within 60 s, the mouse was placed on the platform by the experimenter for 10 s and then placed in a home cage after making them dry with gentle wiping and keeping under a heat lamp for 5 min. On day 5, a probe test was performed where in a 60 s trial the mice swim across the tank without the platform being present. This probe test measures reference memory of the mice as it would look for the platform from its previous memory and spend more time around the original platform location. Video capture and any-maze software were used to analyze data for this experiment. All experiments were performed between 12 p.m. and 4 p.m.

### Vaginal cytology

Estrous cycles were assessed at the same time every day during behavioral study performance day following published protocols [53, 54]. Female mice were properly handled to minimize stress, by gently lifting the animal by the base of tail a plastic pipette filled with about 1 ml of PBS was placed on the tip of the vagina and flushed 5 times with same PBS to allow proper collection of samples for vaginal cytology. Sample was then smeared on appropriate labeled microscope slides and after 1 h of drying time, a crystal violet (0.1%) staining was performed on slides. After drying, the slides were observed under a light microscope to visualize cells. Images were obtained using NIS Elements imaging software version 4.0.

### Statistical analysis

The sample size for the animal study was estimated based on G-power analysis. Test for normality was performed to select the appropriate statistical method. All data are expressed as the mean ± SEM. The values were analyzed by ‘t’ test to compare between two groups (Prism, version 7.0; GraphPad Software Inc., San Diego, CA). P values less than 0.05 were considered statistically significant.

## Results

### Maternal e-cig exposure caused weight reduction in mother and offspring compared to control; offspring plasma confirms presence of nicotine and cotinine

The weight of the mother was measured at gestational day 5 (E5) before starting the e-cig exposure and after delivery. Before starting the treatment (e-cig exposure) at E5, there was no difference in weight between control and treatment group, however after delivery the e-cig exposed mother group had significant weight reduction compared to control group (P < 0.001) (Additional file 1: Fig S1).

**Fig. 2:**
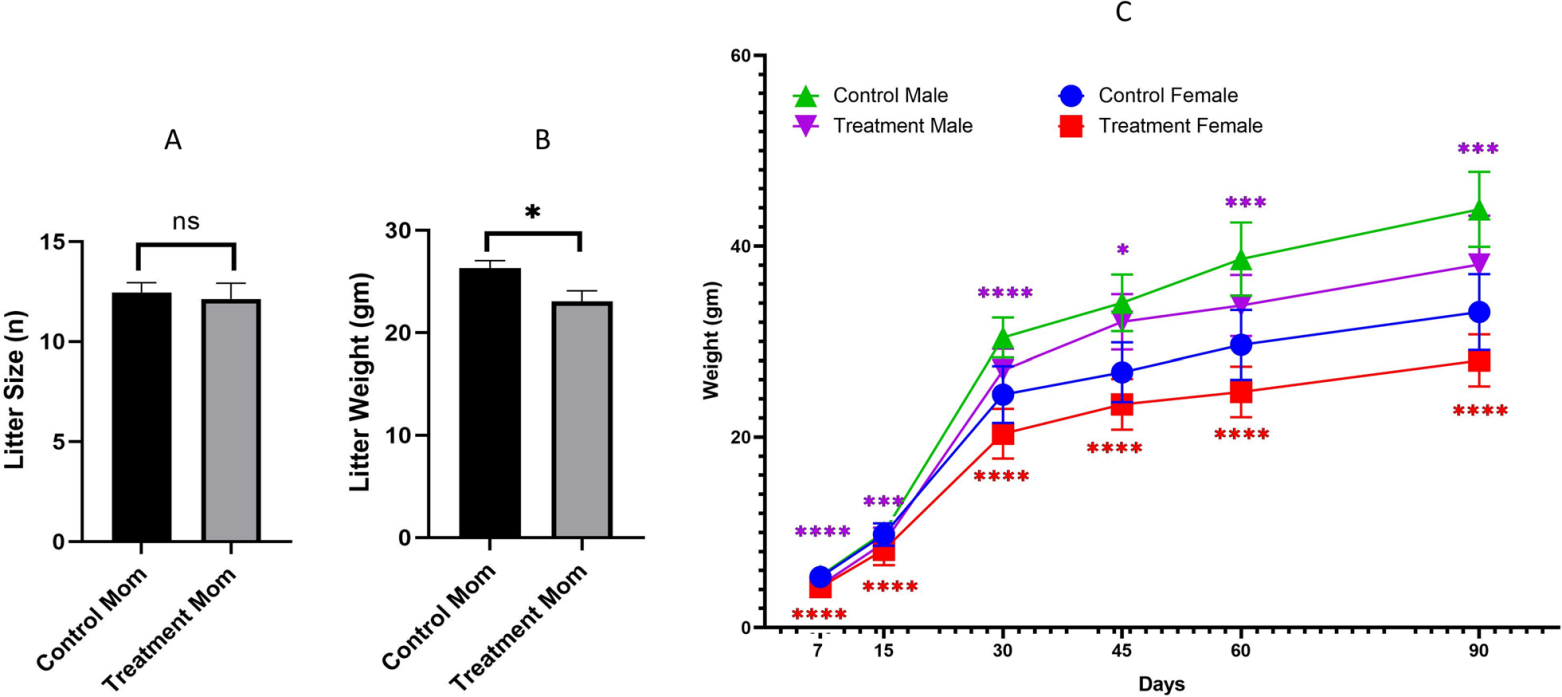
Measurement of **A** litter size and **B** litter weight (n = 8) and **C** offspring body weight at PD7, PD15, PD30, PD45, PD60 and PD90. n = 20–40; *P < 0.05, ***P < 0.001 and ****P < 0.0001

We also counted the litter size after delivery and measured the weight of the offspring at PD0, PD7, PD15, PD30, PD45, PD60, and PD90. No difference was observed in litter size between control and exposed offspring (Fig. 2A), but significantly reduced body weight was observed in prenatally e-cig exposed both male and female offspring at PD0 (P < 0.05), PD7 (P < 0.0001), PD15 (P < 0.0001 for female and P < 0.001 for male), PD30 (P < 0.0001 for both male and female), PD45 (P < 0.0001 for female and P < 0.05 for male), PD60 (P < 0.0001 for female and P < 0.001 for male), and PD90 (P < 0.0001 for female and P < 0.001 for male) compared to control (Fig. 2C).

**Fig. 3:**
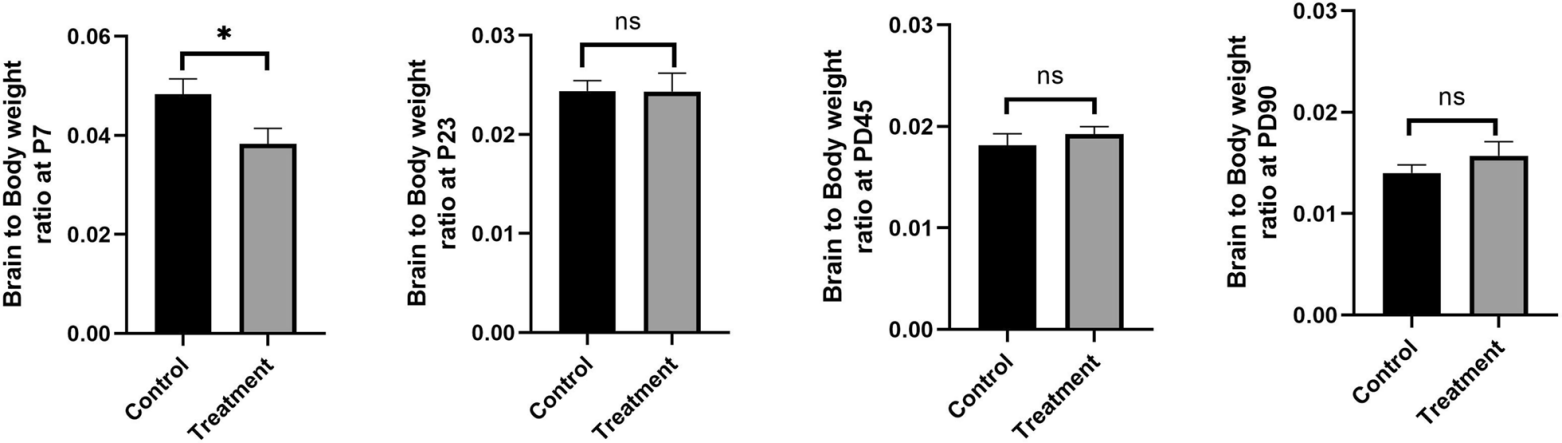
Measurement of brain to body weight ratio of offspring at PD7, PD15, PD30, PD45, PD60 and PD90. n = 10; *P < 0.05

Postnatal brain to body weight ratio was also measured at PD7, PD23, PD45 and PD90. Significantly low brain to body weight ratio was observed in e-cig exposed group at PD7 (P < 0.05) but not at other time points (Fig. 3).

After completing the maternal exposure at PD7, plasma was collected from randomly selected offspring (n = 4) to measure the nicotine and cotinine concentration and we observed 4.3 ± 1.001 ng/mL and 3.4 ± 0.6760 ng/mL of average nicotine and cotinine concentration in plasma respectively (Additional file 2: Fig S2). Previously, our lab also reported the presence of nicotine and cotinine in plasma and brain of offspring after maternal e-cig exposure, the level of which were comparable to those of mother [6].

### Prenatal e-cig exposure alters the expression of BBB component markers

We measured a total of 10 markers which are essential components of the BBB and neurovascular unit including tight junction proteins (ZO-1, claudin-5, occludin), astrocyte (GFAP), pericyte (PDGFR-β), basement membrane protein (laminin α1 and laminin α4), water channel protein (AQP4), glucose transporter (GLUT-1) and neuron specific marker (NeuN) by western blot at PD7, PD23, PD45 and PD90.

#### Tight junction proteins

**Fig. 4:**
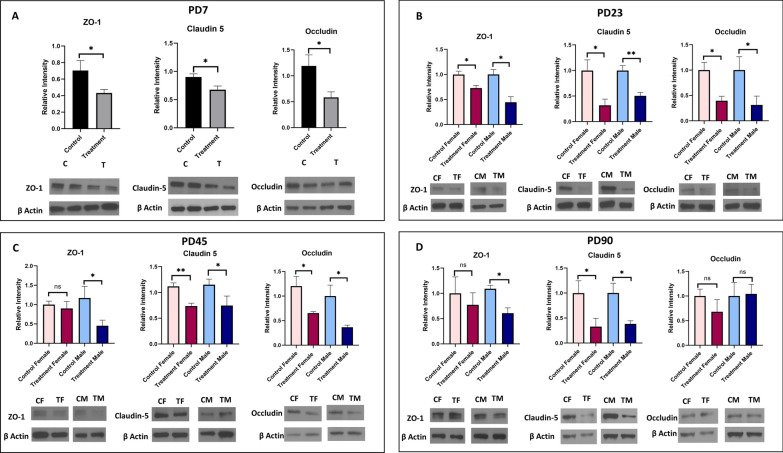
Expression of tight junction proteins ZO-1, claudin-5 and occludin in prenatally e-cig exposed offspring compared to control at **A** PD7, **B** PD23, **C** PD45 and **D** PD90 by western blot; normalized to β-Actin; n = 6 for PD7 and n = 4 for PD23, PD45 and PD90; *P < 0.05, **P < 0.01

Significantly reduced expression of tight junction proteins- ZO-1 (P < 0.05), claudin-5 (P < 0.05), occludin (P < 0.05) was observed at PD7 and PD23 in prenatally e-cig exposed offspring (Fig. 4). Sex-dependent difference was observed at PD45 and PD90 where down regulation of ZO-1 (P < 0.05 for male and no difference for female) was observed in prenatally e-cig exposed male offspring, but not in female (Fig. 4). Significantly reduced expression level of occludin was observed in prenatally e-cig exposed offspring at PD45 (P < 0.05 for both male and female), but not observed at PD90 (Fig. 4). At every time point, significantly reduced expression of claudin-5 (P < 0.05), was found in both male and female prenatally e-cig exposed offspring (Fig. 4).

#### Astrocyte, pericyte, and neuron specific marker

**Fig. 5:**
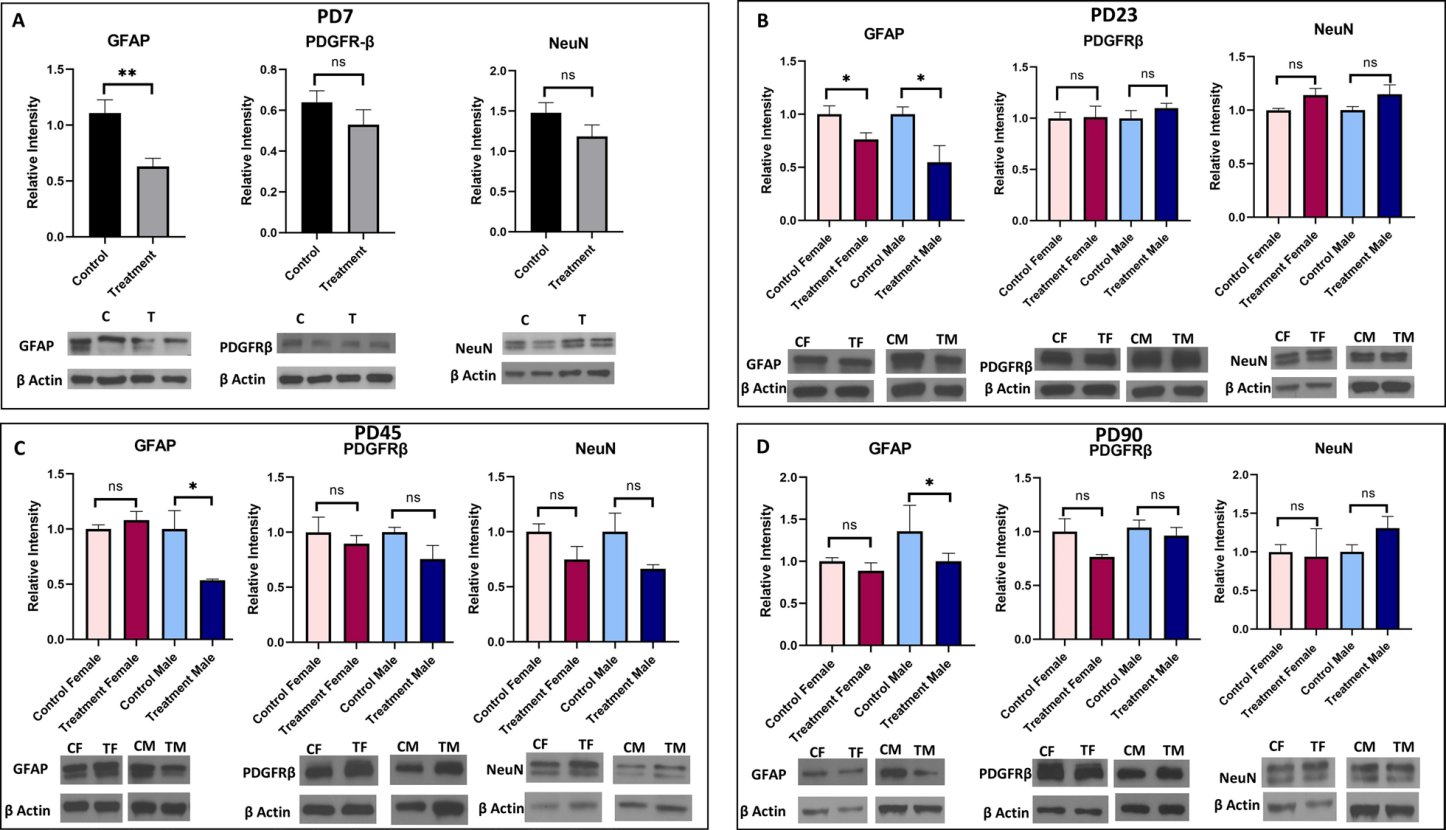
Expression of astrocyte marker (GFAP), pericyte marker (PDGFRβ) and neuron specific marker (NeuN) in prenatally e-cig exposed offspring compared to control at **A** PD7, **B** PD23, **C** PD45 and **D** PD90 by western blot; normalized to β-Actin. n = 6 for PD7 and n = 4 for PD23, PD45 and PD90; *P < 0.05, **P < 0.01

Downregulation of GFAP was observed in prenatally e-cig exposed offspring from PD7 (P < 0.01) to PD90 (P < 0.05) (Fig. 5). Interesting, significantly reduced expression of GFAP was only found in prenatally e-cig exposed male offspring at PD45 (P < 0.05) and PD90 (P < 0.05) (Fig. 5). No difference was observed in PDGFRβ and NeuN expression between control and prenatally e-cig exposed offspring at any time point (Fig. 5).

#### Basement membrane proteins

**Fig. 6:**
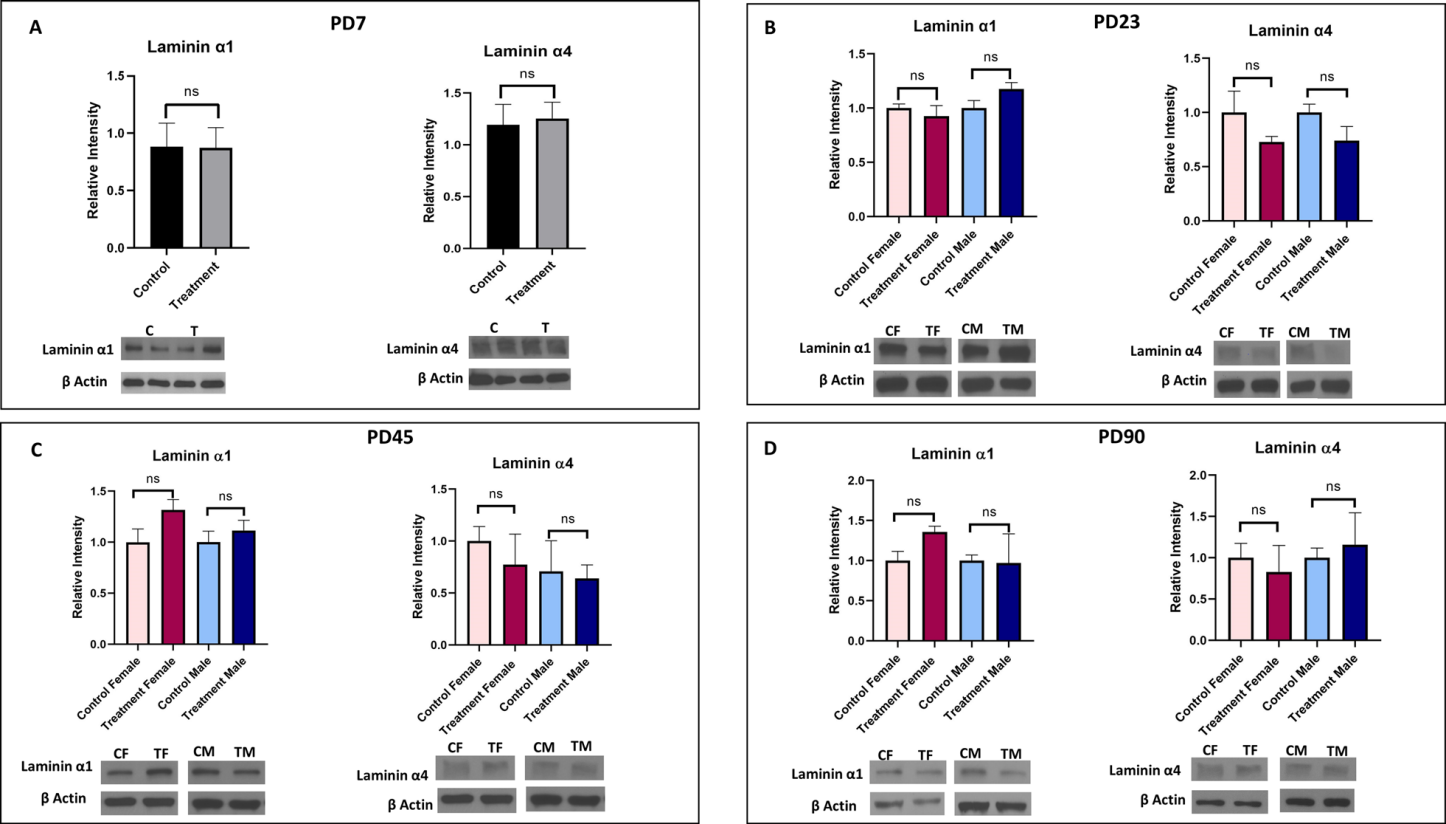
Expression of basement membrane proteins (laminin α1 and laminin α4) in prenatally e-cig exposed offspring compared to control at **A** PD7, **B** PD23, **C** PD45 and **D** PD90 by western blot; normalized to β-Actin. n = 6 for PD7 and n = 4 for PD23, PD45 and PD90; *P < 0.05

Laminin α1 is parenchymal and laminin α4 is endothelial basement membrane protein which are the major components of BBB. In our study, no significant difference was noticed in prenatally e-cig exposed offspring compared to control in any time point (Fig. 6).

#### Glucose transporter and water channel protein

**Fig. 7:**
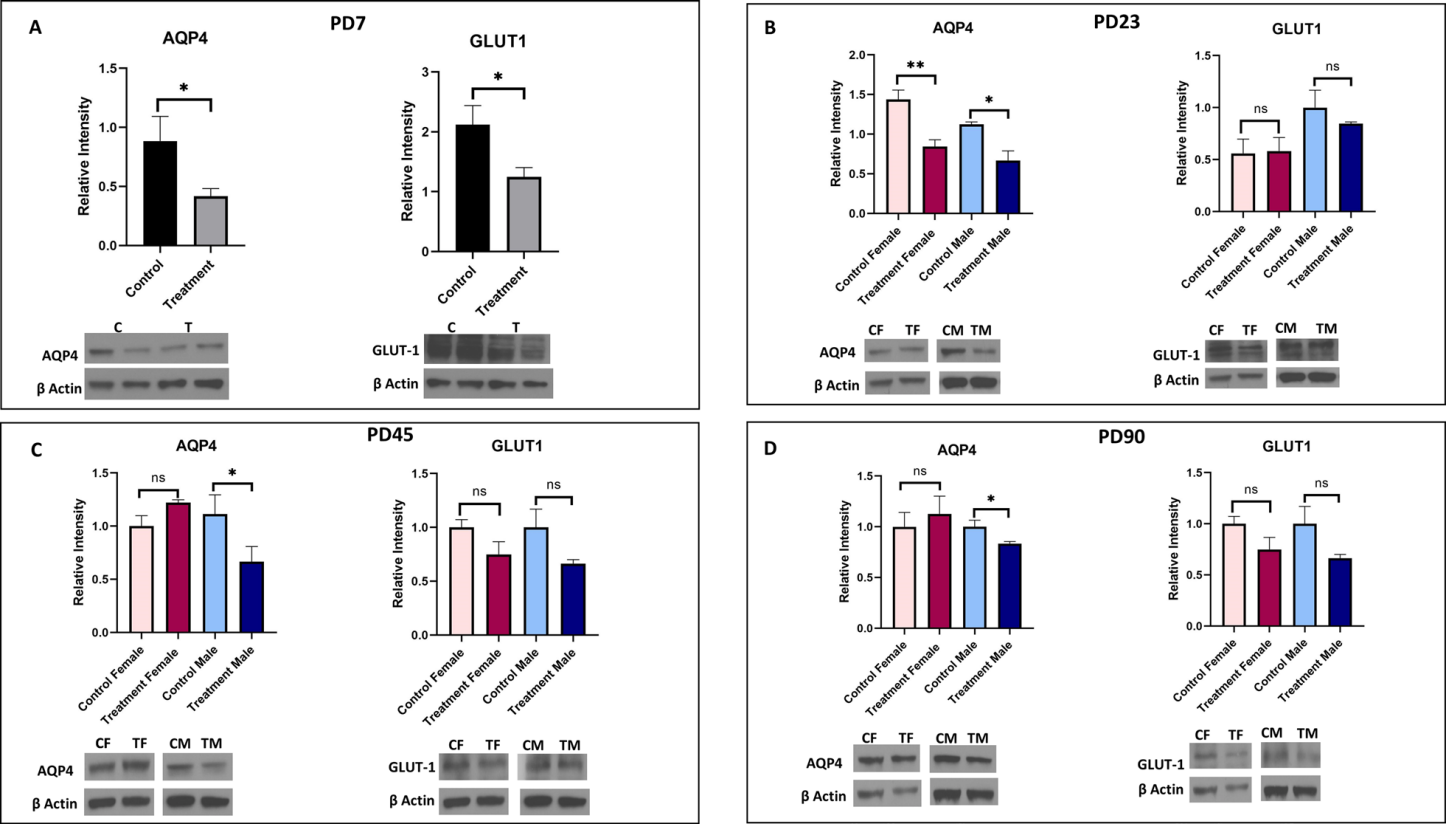
Expression of glucose transporter (GLUT-1) and water channel protein (AQP4) in prenatally e-cig exposed offspring compared to control at **A** PD7, **B** PD23, **C** PD45 and **D** PD90 by western blot; normalized to β-Actin. n = 6 for PD7 and n = 4 for PD23, PD45 and PD90; *P < 0.05, **P < 0.01

Expression of the glucose transporter (GLUT-1) and aquaporin (AQP4) were evaluated in prenatally e-cig exposed offspring and control group at every time point (Fig. 7). Significantly decreased expression of GLUT-1 was observed only at PD7 (P < 0.05). In case of AQP4, downregulation of protein expression was observed at every time point (P < 0.05 and P < 0.01 for female offsprinf at PD23) in prenatally e-cig exposed male and female offspring except for PD45 (P < 0.05) and PD90 (P < 0.05) where decreased expression level was observed only in male (Fig. 7).

**Fig. 8:**
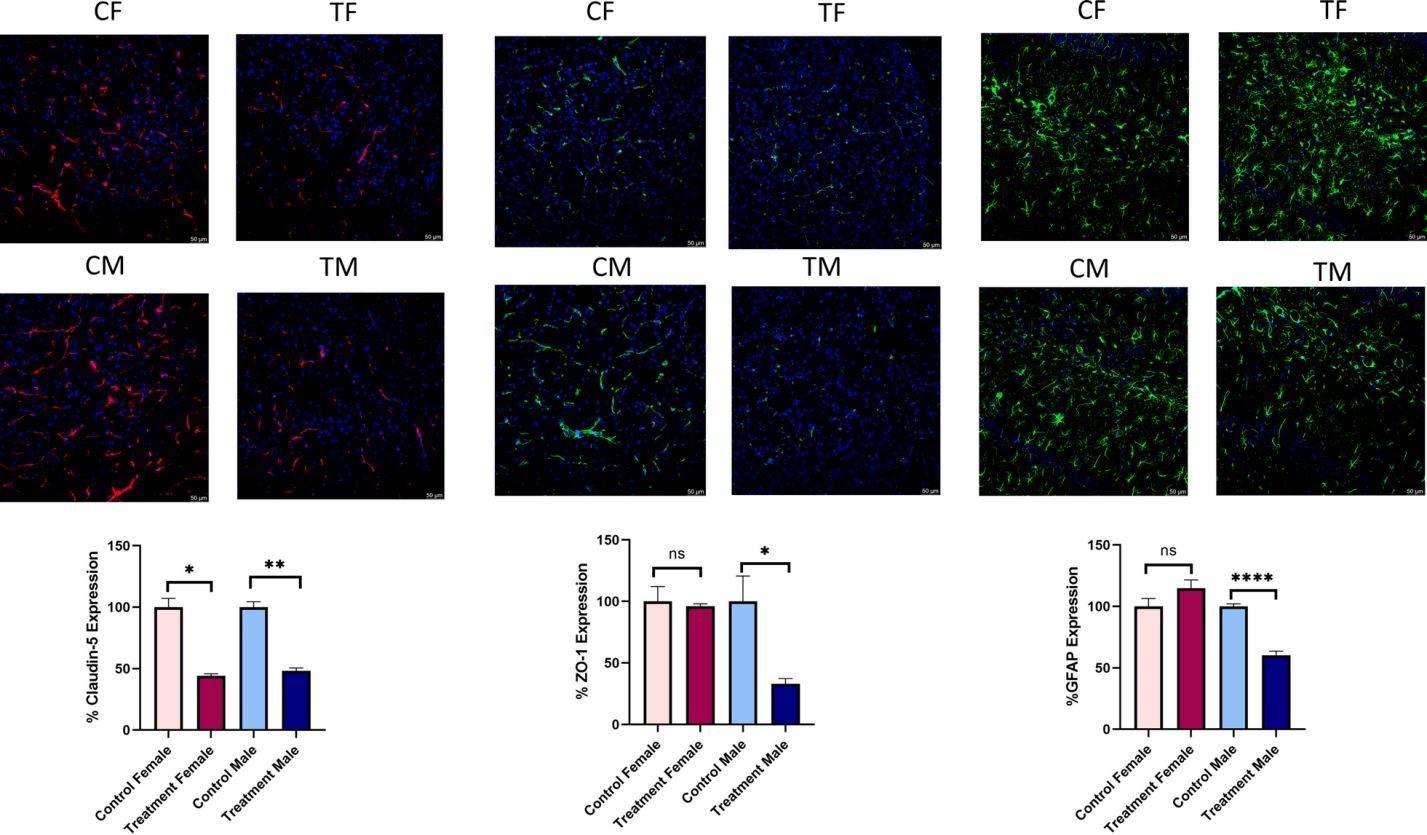
Expression of ZO-1, Claudin-5 (cortex) and GFAP expression (hippocampus) at PD90 in control female (CF), treatment female (TF), control male (CM) and treatment male (TM), normalized to DAPI, n = 3; *P < 0.05, **P < 0.01, ****P < 0.0001

Immunofluorescence: Our immunofluorescence results at PD90 also demonstrated that prenatally e-cig exposed offspring had reduced expression of GFAP, ZO-1 (in male) and Claudin-5 (in both male and female) (Fig. 8).

### Prenatal e-cig exposure causes hyperactivity in female adolescent and adult offspring

**Fig. 9:**
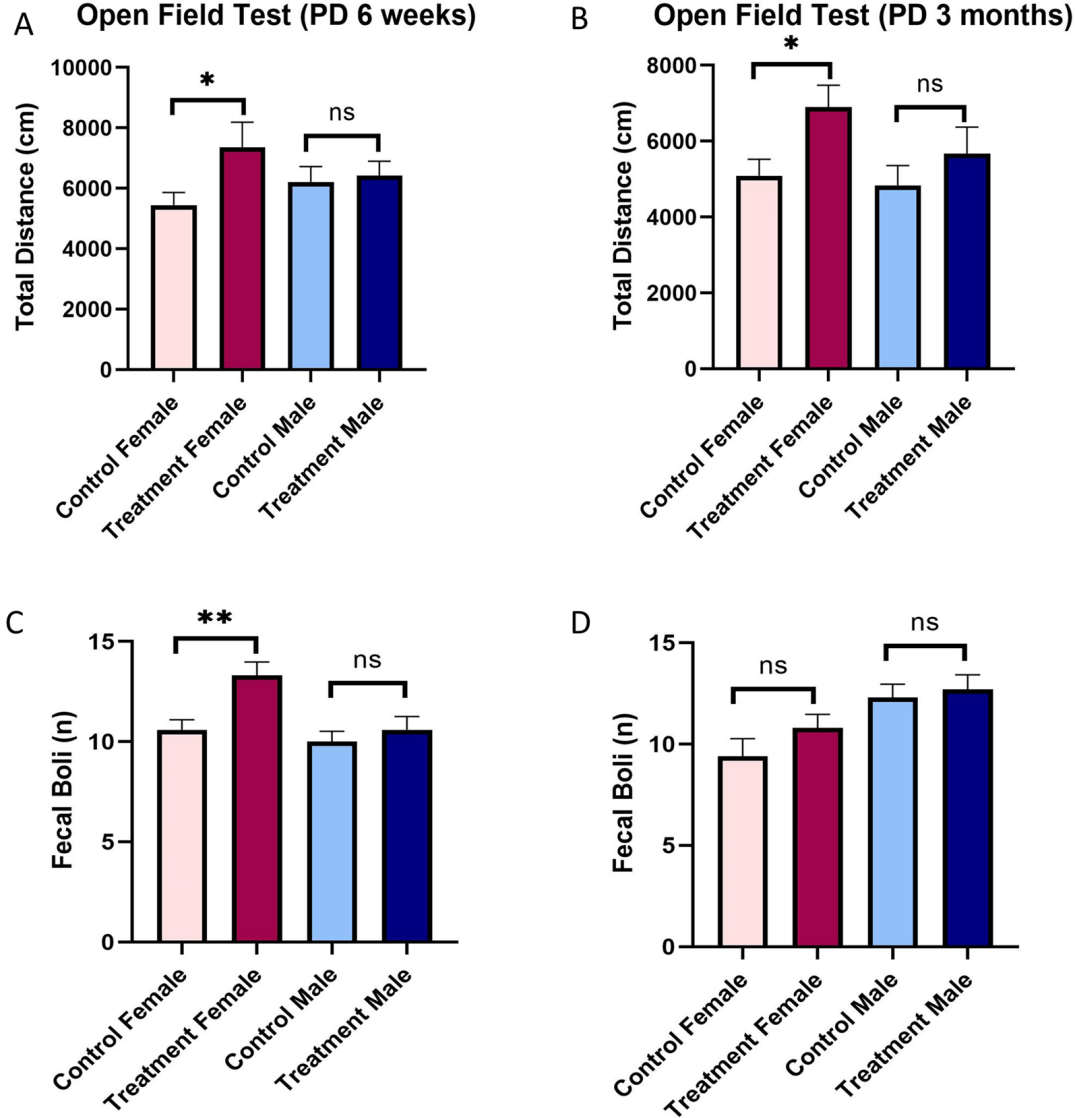
Assessment of locomotor/hyperactivity by open filed test at **A** Adolescence and **B** Adult time point. Fecal boli was counted to evaluate stress/anxiety after OFT at **C** adolescence (PD 6 weeks) and **D** adult (PD 3 months) time point. n = 10; *P < 0.05, **P < 0.01

We measured the locomotor activity of control and prenatally e-cig exposed male and female offspring at their adolescent (PD45) and adult (PD90) time-point. At both time-points, prenatally e-cig exposed female offspring showed significantly higher locomotor activity compared to control counterpart; no significant difference was observed in prenatally e-cig exposed male offspring (Fig. 9A and B. Fecal boli was counted to measure stress/anxiety-like behavior after OFT completion and only prenatally e-cig exposed female offspring had higher fecal boli compared to control at adolescence (Fig. 9C) (P < 0.01) and no difference was observed in adulthood (Fig. 9D).

### Prenatal e-cig exposure deteriorates recognition memory function

**Fig. 10:**
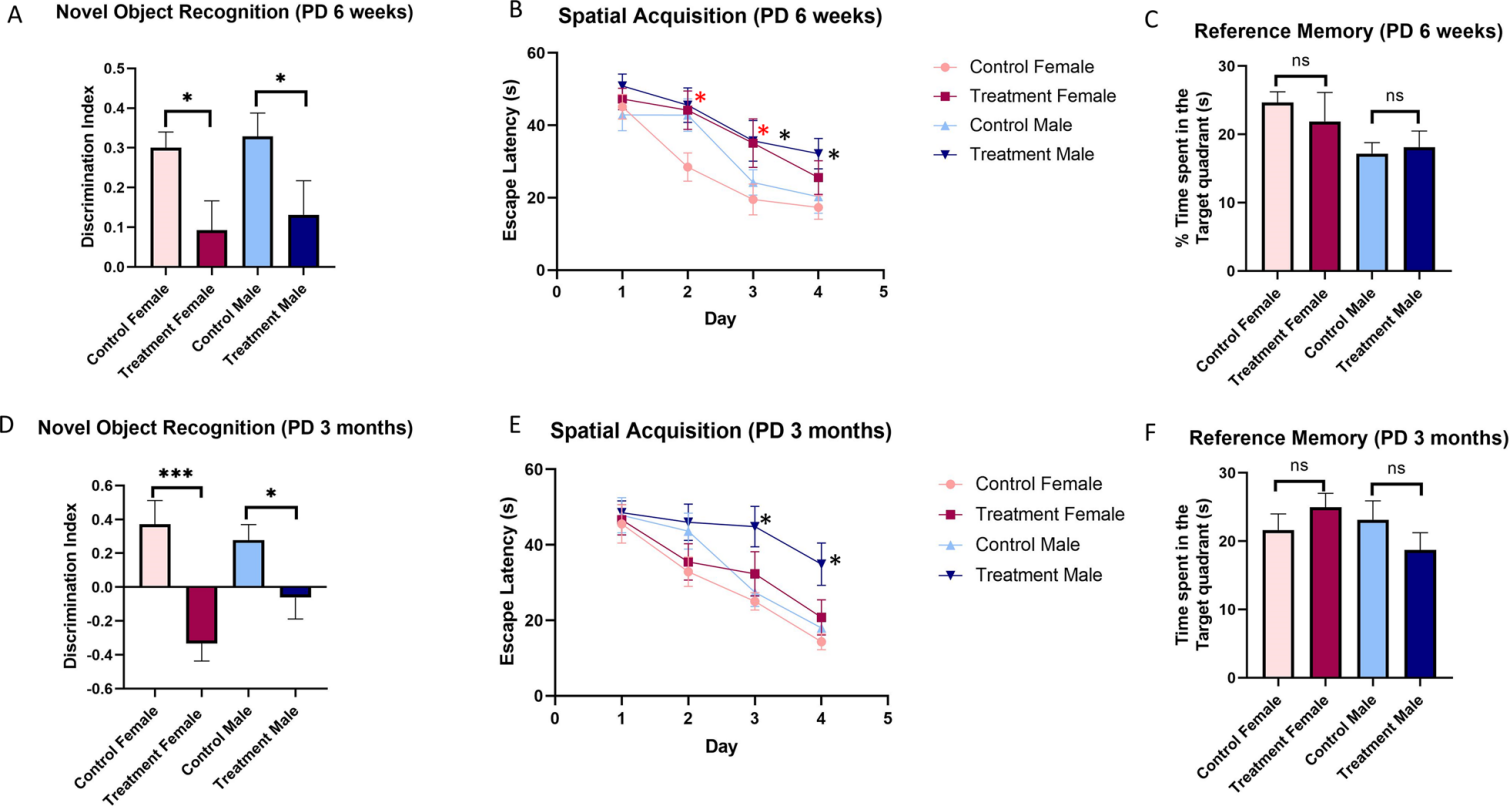
Assessment of learning and memory function in prenatally e-cig exposed offspring compared to control at adolescent and adult age. Novel object recognition test to evaluate short term memory function at **A** PD 6 weeks and **D** PD 3 months; Morris water maze test to assess special learning and memory function at **B**–**C** PD 6 weeks and **E**–**F** PD 3 months, n = 10; *P < 0.05, ***P < 0.001

NORT was conducted to evaluate short-term recognition memory of the adolescent (PD45) (Fig. 10A) and adult offspring (PD90) (Fig. 10D). Prenatal e-cig exposure significantly decreased recognition memory in both prenatally e-cig exposed male and female offspring at (P < 0.05 for both female and male) and adult time-point (P < 0.001 for female and P < 0.05 for male). The results have been shown in Fig. 10A and D.

### Prenatal e-cig exposure did not impact spatial acquisition and reference memory function

MWMT was performed to assess spatial acquisition and reference memory function in offspring at adolescent and adult age. No significant difference was observed in control and treated group (Fig. 10C and F) in reference memory however, prenatally e-cig exposed female and male group had slow learning process (learning the location of hidden platform) compared to control group on day 2–3 and day 3–4 respectively at adolescence (P < 0.05) (Fig. 10B). However, at adulthood only male offspring had impaired learning compared to control at day 3–4 (P < 0.05) (Fig. 10E).

### Reassessment of behavioral outcomes considering estrous cycle in female offspring

We collected vaginal secretions from offspring to evaluate the impact of estrous cycle on behavioral outcomes. Vaginal secretions are made up of three types of cells- leucocytes, anucleated cornified epithelial cells and nucleated epithelial cells. Estimation of the phase of estrous cycle is based on the proportion of these cells in the vaginal secretion. In proestrus stage, the proportion of nucleated epithelial cell is high whereas in estrus stage, cornified cells are abundant as you can see in Additional file 3: Fig S3. In metestrus stage, three types of cells are present and in diestrus stage, the prominent cell is the leukocyte (Additional file 3: Fig S3). In proestrus and estrus stages, the circulating estradiol level is high and in metestrus and diestrus stages, the circulating estradiol level is low. We reassessed open field test (Additional file 3: Fig S3A) and novel object recognition test (Additional file 3: Fig S3B) and observed that circulating estradiol level depending on estrous stages does not impact behavioral outcomes.

## Discussion

In recent years, e-cigs have become extremely popular among all age and sex groups, including pregnant women. The health impact of e-cig vaping is currently unclear, hence it should not be considered as a safe alternative to tobacco smoking during pregnancy as e-cig contains several toxic compounds including propylene glycol, vegetable glycerin, formaldehyde, acrolein, flavoring chemicals, and other trace elements, some of which may be neurotoxic to the developing fetus and offspring [14]. In addition to these chemicals, several studies have demonstrated that in-utero exposure of nicotine and tobacco smoke may impact neonatal brain development which can result in neuro-developmental abnormalities, including increased cell density in the hippocampus [55], reduced volumes of cortical gray matter [56], increased sensitivity to neonatal HI brain injury [8, 51], abnormalities in cell differentiation, disruption in neurotransmitter activity and neurobehavioral dysfunction [57]. Interestingly, post-birth exposure to nicotine did not show similar adverse effects suggesting differences in biological mechanisms during the gestation phase [57]. Although the neurotoxic effect of prenatal nicotine and tobacco exposure is well documented and well established, only a handful of studies have investigated the potential neurotoxic effects of prenatal e-cig exposure on neonates, mostly focusing on cognitive dysfunction [58, 59]. Previously, our lab published that maternal e-cig exposure is associated with decreased brain glucose utilization and worsened hypoxic–ischemic brain injury in offspring [6]. However, no study to date has looked at the effects of prenatal e-cig exposure on BBB integrity which is a critical determinant of cerebrovascular dysfunction in adulthood. In this study, we provided in-vivo evidence that maternal e-cig exposure disrupts some of the critical elements of BBB integrity and deteriorated motor, learning and memory function in male and female offspring at different stages of life.

In our study, we have investigated all the crucial markers for BBB integrity including tight junction proteins (ZO-1, claudin-5, occludin), astrocyte marker (GFAP), pericyte marker (PDGFRβ), neuron specific marker (NeuN), basement membrane protein (laminin α1, laminin α4), glucose transporter (GLUT-1) and water channel protein (AQP4) at different time points (PD7, PD23, PD45 and PD90) (Table 1). We have observed that prenatally, e-cig exposed offspring had reduced expression of tight junction proteins (mainly ZO-1 and claudin-5) until PD90. These results are consistent with previous literature demonstrating the downregulation of tight junction proteins mediated by nicotine and tobacco smoke [60–63]. Interestingly in our study, sex-dependent effect has been observed in TJ protein expression in adolescent and adult age. Prenatally, e-cig exposed male adolescent and adult mice had reduced expression of ZO-1 at PD45 and PD90 whereas reduced level of claudin-5 was observed in both male and female offspring at every time point. Claudin-5 is one of the important components of tight junction strand, particularly in endothelial cells of brain which selectively restricts the transport of ions and macromolecules through the tissue barrier [64–66]. Several studies have demonstrated that claudin-5 plays a pivotal role in BBB permeability and downregulation of claudin-5 may result in disrupted brain function [67, 68] by enhancing the passage of macrophages, leukocytes, endotoxins, bacteria, and drugs from the peripheral circulation into the brain [69]. Our study demonstrated decreases in claudin-5 expression in prenatally e-cig exposed male and female offspring at all time points which could be a concern in postnatal health considering its important role in BBB integrity and permeability. Decreased expression of occludin was also observed in prenatally e-cig exposed both male and female offspring in every time point except adulthood (PD90). Sex-dependent impact on BBB integrity and permeability have been reported in several studies. The iPSC-derived BMECs from pre-menopausal women had reduced permeability, and increased barrier strength, compared to iPSC-derived BMECs from men [70]. Increased mRNA expression of tight junction proteins (claudin-1, claudin-5, claudin-12, occludin, ZO-1), junction adhesion molecule A (JAMA), major facilitator superfamily domain containing 2, and brain-derived neurotrophic factor (BDNF) were also observed in female mice compared to male [71]. Considering the biological significance of sex-differences in BBB integrity and permeability, sex dependent BBB characteristics should be further investigated capitalizing on different in-vitro and in-vivo models to more precisely determine the role of sex as a biological determinant of neurovascular function in cerebrovascular diseases [72].

**Table 1:**
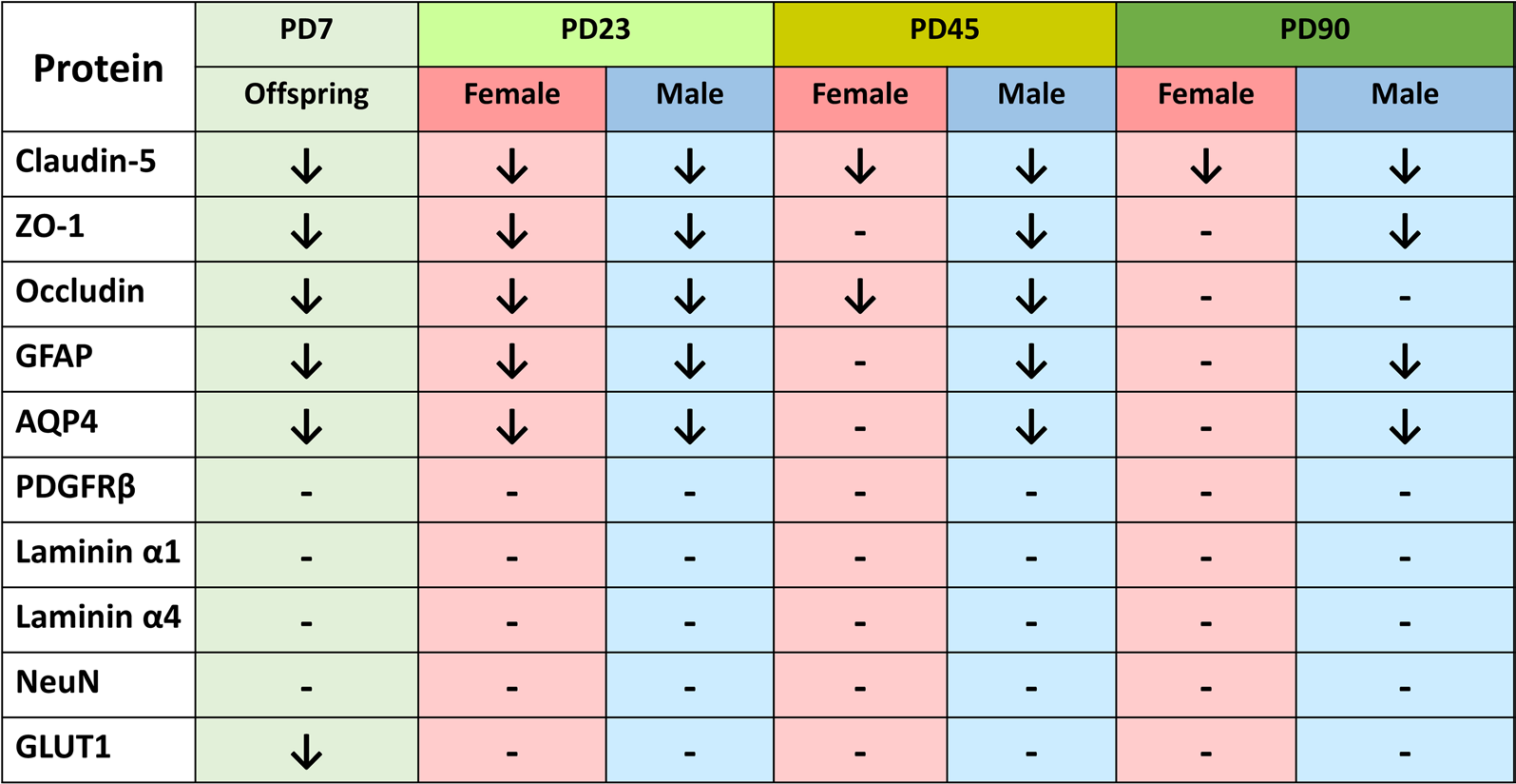
Summary of changes in BBB integrity markers in prenatally e-cig exposed offspring at PD7, PD23, PD45 and PD90

Glial fibrillary acidic protein or GFAP is astrocyte specific marker. Astrocytes facilitate the formation of complex neocortical circuitries involving a complex process of synaptogenesis, maturation, and synaptic pruning in the developing brain [73, 74]. Studies have shown that loss of GFAP expression results in enhanced susceptibility to ischemic insult, increased hippocampal LTP, reduced cerebellar long-term depression (LTD) and decreased glutamate transport [75]. Aquaporin 4 (AQP4) is a water channel protein which is highly expressed on astrocytic endfeet in the brain and plays a pivotal role in modulating astrocytic function, regulating extravascular brain water, brain volume homeostasis, synaptic plasticity as well as producing cerebrospinal fluid. Studies have confirmed the association of AQP4 in the pathophysiology of cerebral disorders including but not limited to stroke, cerebral edema, traumatic brain injury, Parkinson's disease, epilepsy, and depression [76]. In our study, we have observed downregulation of GFAP and AQP4 expression at every time point in prenatally e-cig exposed offspring till adulthood which indicates a potential neurotoxic effect of prenatal e-cig exposure. Interestingly, downregulation of GFAP and AQP4 was only observed in male offspring at PD45 and PD90 which indicates a sex difference in GFAP and AQP4 expression. Studies have reported the association of sex hormone with astrocytes expression. Estradiol (E2) has been reported as a mediator of neuronal sprouting through its effects on astrocytes [77], and it regulates the expression of GFAP, the major intermediate filament protein of differentiated astrocytes, both in-vitro and in-vivo model in the hypothalamus and the hippocampus of the rat [78, 79]. Arias C et al. reported higher expression of GFAP in CA1, CA3, and dentate gyrus in proestrus females as compared with males and diestrus females suggesting the association of sex steroid hormones in the sexually dimorphic functions of the hippocampus, and changes in its activity during the estrous cycle. In our study, we have also observed the downregulation of GFAP in prenatally e-cig exposed male offspring, not in female in adolescent and adult time point indicating a role of sex steroid hormones in GFAP expression. AQP4 is a water channel protein which is essential in maintenance of cerebral water balance and highly expressed in astrocytic end foot at BBB [80]. In our study, we have observed downregulation of AQP4 similar to GFAP. In PD45 and PD90, significantly lower expression level of AQP4 was observed in prenatally e-cig exposed male offspring, but not in female. As AQP4 is abundantly expressed in astrocytic end foot, downregulation of GFAP may have resulted in lower expression of AQP4 in prenatally e-cig exposed male offspring in our study.

We also evaluated the expression of basement membrane proteins, laminin α1 (parenchymal) and laminin α4 (endothelial), pericyte marker (PDGFRβ) and neuron specific marker (neuN) in our study however we did not observe any significant difference between prenatally e-cig exposed offspring and control offspring in these proteins’ expression in offspring brain.

Moreover, we assessed the effect of maternal vaping on long-term locomotor, learning and memory functions of offspring at adolescent and adult stage. OFT measures the hyperactivity or locomotor activity in rodent model. Hyperactivity is one of the elements of attention deficiency hyperactivity disorder (ADHD) in children [81]. Several studies have demonstrated the association between maternal smoking and ADHD in offspring [82–84]. In our study we have observed that prenatally e-cig exposed female offspring had higher locomotor activity than control group at adolescence and adulthood. However, no difference has been observed in prenatally e-cig exposed and control male group. This result is consistent with previous finding where higher locomotor activity was observed in female rats compared to male rats at low dose of nicotine injection [85]. Another study demonstrated the anxiogenic response in female mice treated with chronic nicotine and no effect was observed in males [86]. A recent study also reported that female offspring with maternal nicotine exposure demonstrated an increase in anxiety-like behavior in open-field test which is consistent with our findings [87]. These studies clearly indicate the potential role of sex in differential hyperactivity between males and females. Our study also demonstrated a sex-difference in hyperactivity followed by prenatal e-cig exposure in male and female offspring at PD45 and PD90. We also measured defecation or fecal boli counting after OFT and prenatally e-cig exposed female offspring had significantly higher fecal boli at adolescence demonstrating higher stress/anxiety-like behavior compared to control group.

We evaluated short-term memory function by NORT, where decreased recognition memory was observed in prenatally e-cig exposed both male and female offspring at adolescence and adulthood. MWMT was performed to evaluate spatial learning and memory function in offspring. No significant difference has been observed in reference memory function in prenatally exposed offspring at adolescence and adult time points. However, prenatally e-cig exposed male and female offspring at PD45 and only male offspring at PD90 had worsened spatial acquisition measured by escape latency. These results indicate that prenatal e-cig exposure worsens learning and memory function in adolescent and adult offspring. These results are consistent with some of the previously published literature investing exposure to tobacco smoke. Impaired cognitive development and lower intelligence quotient were observed in prenatally tobacco smoke exposed offspring [88, 89]. A recent literature review has demonstrated that children prenatally exposed to smoking are more likely to require support resulting from the well-documented physical, socio-emotional, behavioral, mental, and neurocognitive consequences of exposure [90]. Interestingly, maternal e-cig exposure has been found to be associated with short-term memory deficits, reduced anxiety, and hyperactivity in adult offspring using novel object recognition and elevated plus maze tests [58]. A recent study from our lab also demonstrated that maternal e-cig exposure resulted in cognitive deficits in e-cig exposed offspring with hypoxic-ischemic (HI) brain injury compared to sham offspring group [6].

Sex differences have been observed in pharmacokinetics and pharmacodynamics of nicotine in several studies [72]. Decreased nicotine clearance and lower ratio of nicotine/cotinine was reported in women compared to men due to faster metabolism of nicotine [6, 91]. Previous study has also shown differential responses of cerebral cortex to nicotine in male and female [92]. Similarly, our study has also found sex-specific effect of nicotine containing e-cig on BBB and behavioral outcomes and these results might be explained by sex differences in nicotine metabolism.

To evaluate the role of estradiol in behavioral outcomes, we re-assessed the data based on estrous cycle. Estrous cycle in mice consists of 4 stages: Proestrus, estrus, metestrus and diestrus. In proestrus and estrus days, circulating estradiol level is high and in metestrus and diestrus days, the circulating estradiol level is low [53]. Studies have demonstrated a sex-dependent effect of nicotine in locomotor activity. Higher locomotor activity was observed in female rats compared to male rats at low dose of nicotine injection [93]. However, another study reported contradictory results demonstrating higher locomotor activity in adolescent males compared to females after single dose nicotine administration with minipumps [94]. Lower dose of nicotine administration was also reported to cause decreased locomotor activity in female than male, using osmotic mini pumps [95]. Even though the nicotine-exposed adolescents demonstrated contradictory results, it is apparent that females are more sensitive to the nicotine induced locomotor activity compared to male, and possibly, ovarian hormones play a role in this greater responsivity. Interestingly, in our study we did not find the role of estradiol level in behavioral outcomes (OFT and NORT). Therefore, future experimental design should focus on acute vs. chronic nicotine dosing through vaping when interpreting effects on locomotor activity.

## Conclusion

Tobacco smoking during pregnancy is well documented as one of the crucial preventable causes of adverse birth outcomes across the world while the widespread use of recently introduced e-cig products among pregnant women poses additional challenges due to limited studies. E-cig is considered as a safe alternative to conventional cigarette and has become a pressing issue regarding its safety. The safety issue is even more salient during pregnancy as maternal e-cig use can be a threat to postnatal health due to presence of nicotine and several known and unknown additives in e-cig liquid. Considering the prevalence of e-cig use during pregnancy and associated health risks, the aim of our study was to evaluate the long-term impact of maternal e-cig use on postnatal BBB integrity and behavioral outcomes in *in-vivo* model. In this study, we have shown that maternal e-cig exposure can decrease the expression of tight junction proteins, astrocyte marker and AQP4 at PD7, PD23, PD45 and PD90. We also observed sex-different effect in case of ZO-1, GFAP and AQP4 expression at PD45 and PD90. However, no difference was observed in case of PDGFRβ, laminin (α1, α4) and NeuN expression at any time point. Consistent reduction of tight junction protein, claudin-5 till PD90 in both prenatally e-cig exposed male and female offspring is a serious health concern as it could be associated with long term neurotoxicity and cerebrovascular diseases. In our study we have also observed low birth weight and significantly reduced body weight in prenatally e-cig exposed male and female offspring till PD90. Considering that low birth weight is associated with neurodevelopmental deficits, the combined effect of lower body weight and reduced BBB integrity could indicate the severity of prenatal e-cig toxicity on postnatal health including both sexes. Moreover, this study has shown that maternal e-cig exposure deteriorates motor, learning and memory function in adolescent and adulthood. As disruption of key components of BBB integrity can result in worsened cerebral and neurological dysfunction including ischemic stroke, further studies are warranted to evaluate the impact of maternal vaping on the pathogenesis of different cerebrovascular diseases in adult and aged offspring.

## Supplementary Information

**Additional file 1: Figure S1.** Measurement of mother weight at (A) E5 (before exposure) and (B) post-delivery (after exposure), n=8. ***P<0.001

**Additional file 2: Figure S2.** Measurement of plasma nicotine and cotinine concentration in prenatally e-cig exposed offspring at PD7; plasma nicotine and cotinine concentration are 4.3 ng/mL and 3.4 ng/mL respectively, n=4.

**Additional file 3: Figure S3.** Re-assessment of behavioral outcomes in prenatally e-cig exposed offspring compared to control at PD90 considering estrous cycle. (A) Open field test to measure locomotor/hyperactivity; (B) Novel object recognition test to evaluate short term memory function. n=4-6; *P< 0.05

## Data Availability

All data generated or analyzed during this study are included in this published article (and its Additional files).

## Abbreviations

ENDS: Electronic nicotine delivery system
E-cig: Electronic cigarette or e-cigarette
BBB: Blood-brain barrier
E5: Gestational day 5
PD7: Postnatal day 7
PD15: Postnatal day 15
PD23: Postnatal day 23
PD30: Postnatal day 30
PD45: Postnatal day 45
PD60: Postnatal day 60
PD90: Postnatal day 90
LBW: Low birth weight
SIDS: Sudden infant death syndrome
CNS: Central nervous system
EC: Endothelial cell
CORESTA: Cooperation centre for scientific research relative to tobacco
BCA: Bicinchoninic acid
TBST: Tris-buffered saline
OFT: Open field test
NORT: Novel object recognition test
MWMT: Morris water maze test
JAMA: Junction adhesion molecule A
BDNF: Brain-derived neurotrophic factor
E2: Estradiol
ADHD: Attention deficiency hyperactivity disorder
HI: Hypoxic-ischemic
LTD: Long-term depression
GFAP: Glial fibrillary acidic protein
AQP4: Aquaporin 4
