# Supplementary figures and images for: Maternal e-cigarette use can disrupt postnatal blood-brain barrier (BBB) integrity and deteriorates motor, learning and memory function: influence of sex and age
Source: Fluids Barriers CNS. 2023 Mar 10;20:17. doi: 10.1186/s12987-023-00416-5 (PMC9999561; doi:10.1186/s12987-023-00416-5)

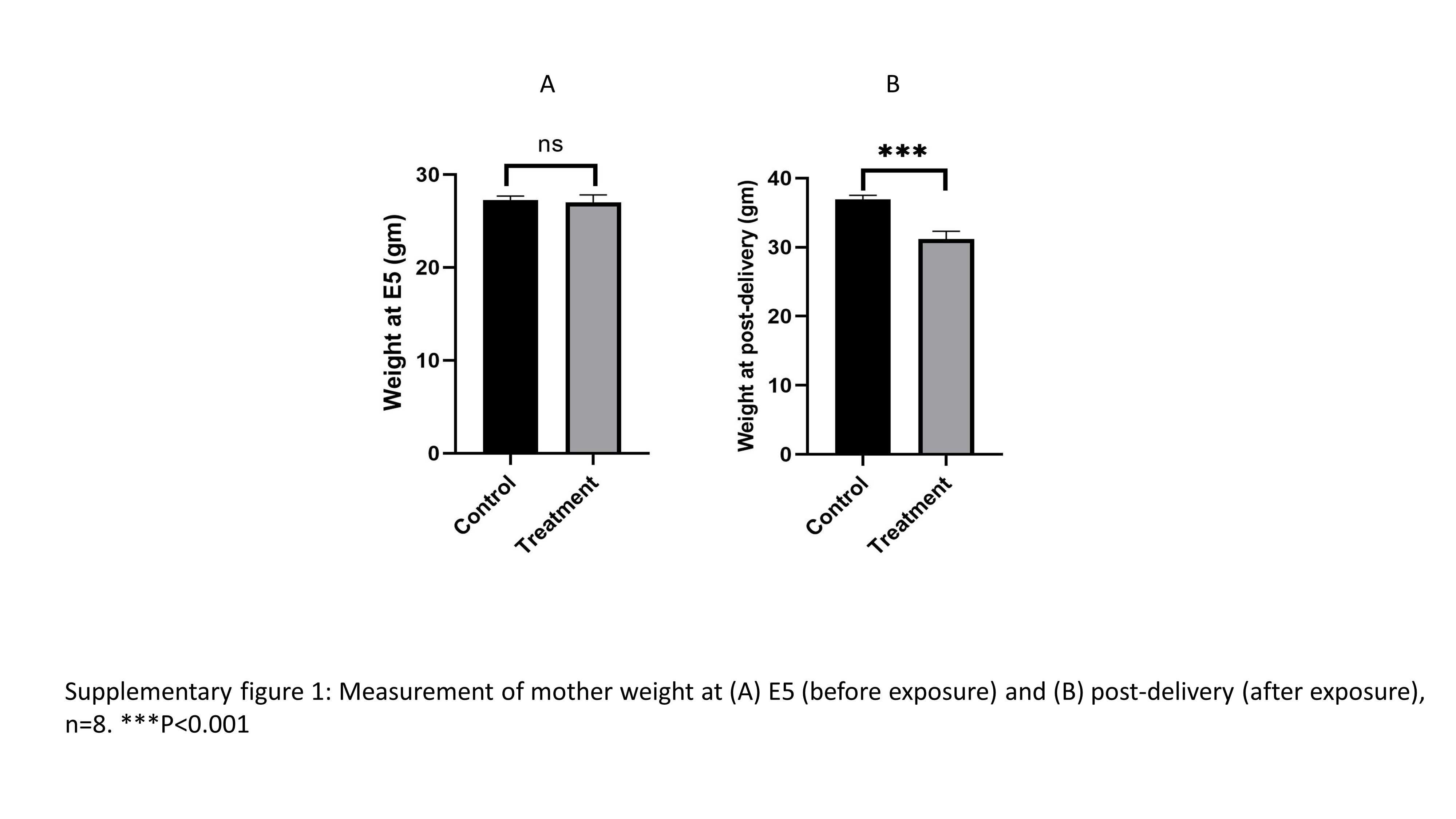

Supplement: Supplementary file 1 — Additional file 1: Figure S1. Measurement of mother weight at (A) E5 (before exposure) and (B) post-delivery (after exposure), n=8. ***P<0.001 [file 12987_2023_416_MOESM1_ESM.jpg]

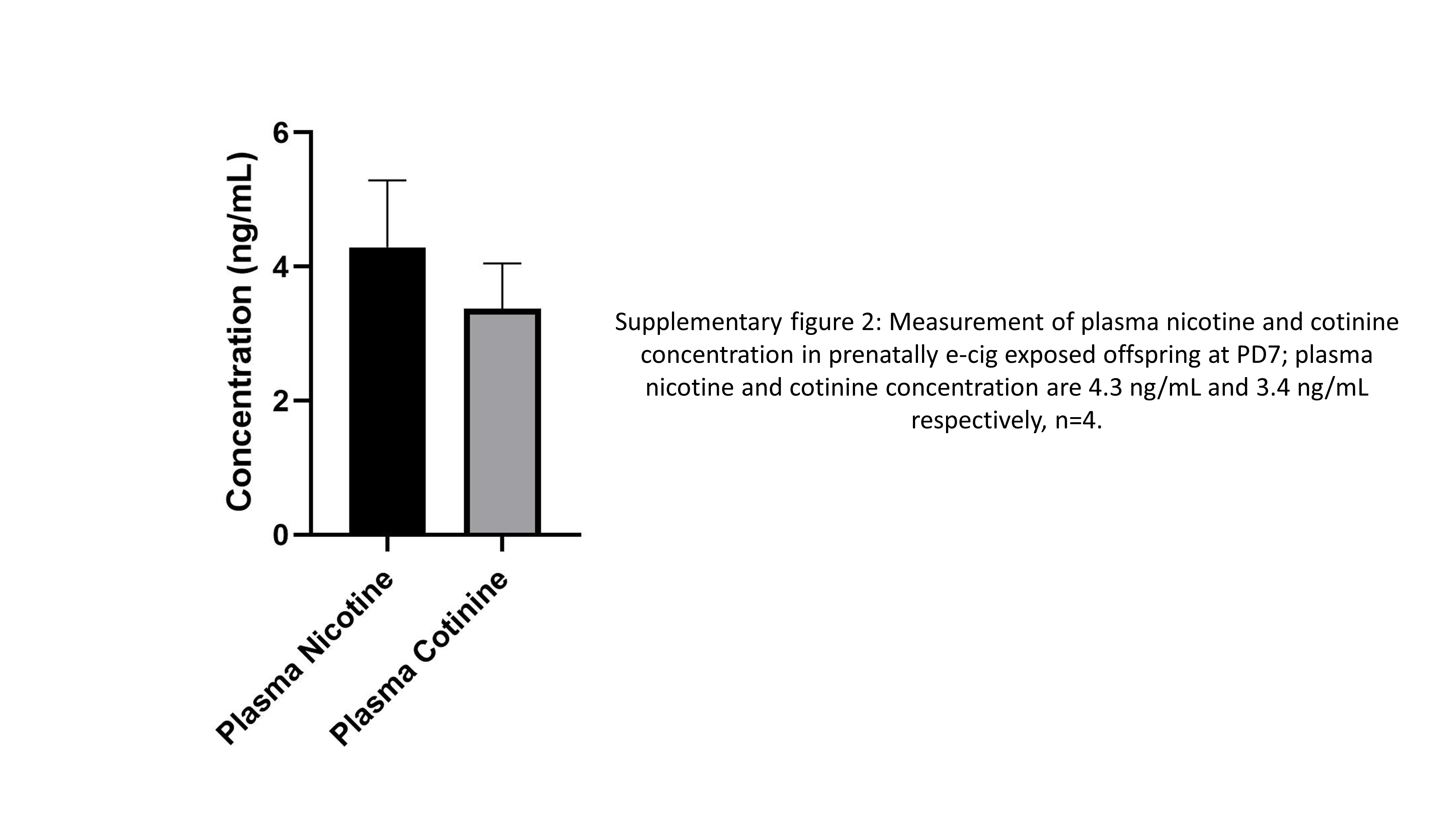

Supplement: Supplementary file 2 — Additional file 2: Figure S2. Measurement of plasma nicotine and cotinine concentration in prenatally e-cig exposed offspring at PD7; plasma nicotine and cotinine concentration are 4.3 ng/mL and 3.4 ng/mL respectively, n=4. [file 12987_2023_416_MOESM2_ESM.tif]

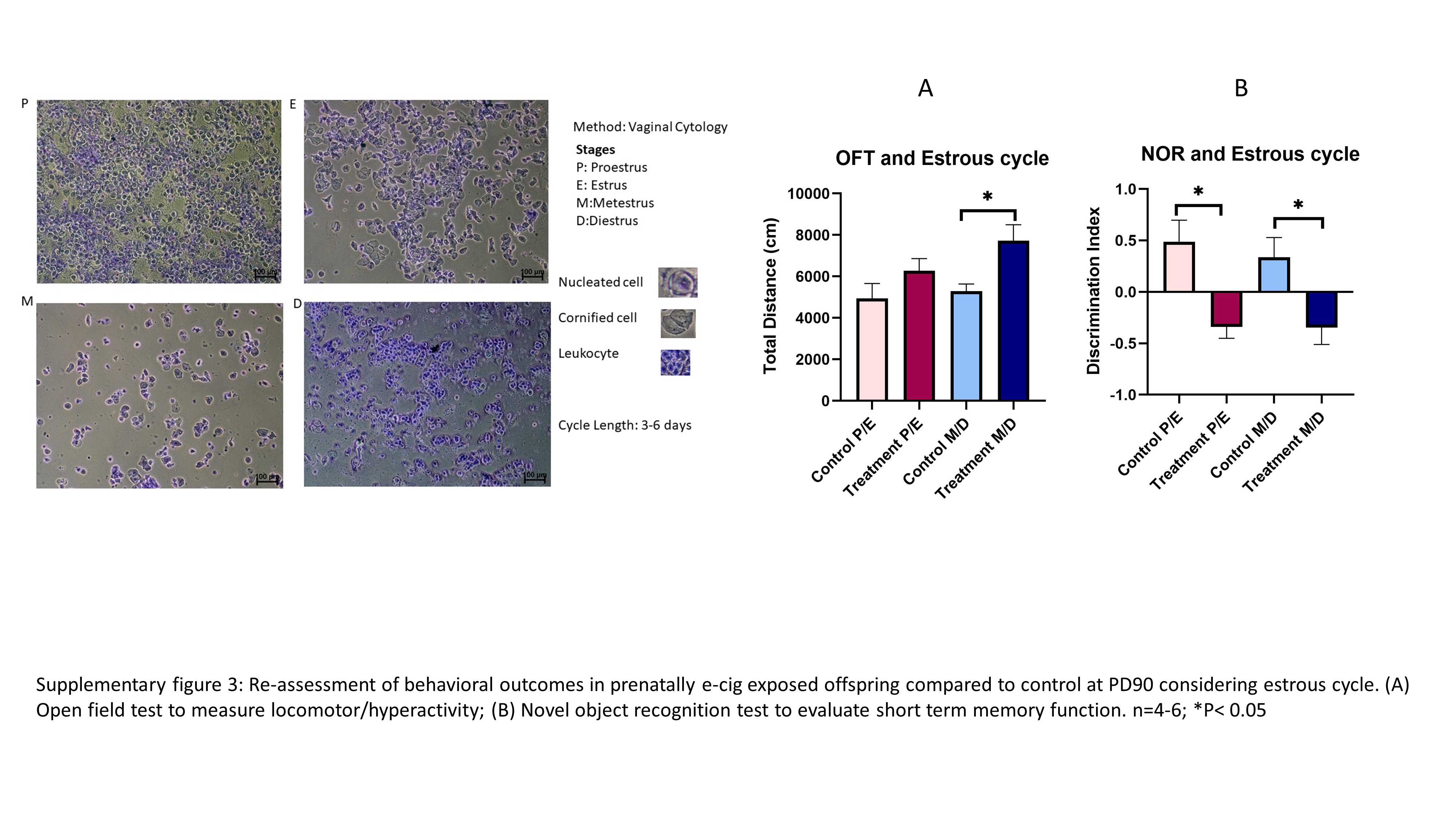

Supplement: Supplementary file 3 — Additional file 3: Figure S3. Re-assessment of behavioral outcomes in prenatally e-cig exposed offspring compared to control at PD90 considering estrous cycle. (A) Open field test to measure locomotor/hyperactivity; (B) Novel object recognition test to evaluate short term memory function. n=4-6; *P< 0.05 [file 12987_2023_416_MOESM3_ESM.tif]
